# Pancreatic Neuroendocrine Tumor: The Case Report of a Patient with Germline *FANCD2* Mutation and Tumor Analysis Using Single-Cell RNA Sequencing

**DOI:** 10.3390/jcm13247621

**Published:** 2024-12-14

**Authors:** Ekaterina Avsievich, Diana Salimgereeva, Alesia Maluchenko, Zoia Antysheva, Mark Voloshin, Ilia Feidorov, Olga Glazova, Ivan Abramov, Denis Maksimov, Samira Kaziakhmedova, Natalia Bodunova, Nikolay Karnaukhov, Pavel Volchkov, Julia Krupinova

**Affiliations:** 1 Moscow Clinical Scientific Center N.A. A.S. Loginov, Moscow 111123, Russia; e.avsievich@mknc.ru (E.A.); d.salimgereeva@mknc.ru (D.S.); m.v.voloshin@mknc.ru (M.V.); i.feidorov@mknc.ru (I.F.); ol.glazova@gmail.com (O.G.); abriv@bk.ru (I.A.); n.bodunova@mknc.ru (N.B.); n.karnaukhov@mknc.ru (N.K.); vpwwww@gmail.com (P.V.); 2 Moscow Center for Advanced Studies, Kulakova Street 20, Moscow 123592, Russia; alesyamaluchenko@gmail.com (A.M.); zoya.antysheva@gmail.com (Z.A.); maksimov.denis.o@gmail.com (D.M.); skaziahmedova@gmail.com (S.K.); 3 Federal Research Center for Innovator, Emerging Biomedical and Pharmaceutical Technologies, Moscow 125315, Russia

**Keywords:** pancreatic neuroendocrine tumors, pancreatic PanNETs, WES, germline mutation, *FANCD2*

## Abstract

**Background:** Neuroendocrine neoplasms are a rare and heterogeneous group of neoplasms. Small-sized (≤2 cm) pancreatic neuroendocrine tumors (PanNETs) are of particular interest as they are often associated with aggressive behavior, with no specific prognostic or progression markers. Methods: This article describes a clinical case characterized by a progressive growth of nonfunctional PanNET requiring surgical treatment in a patient with a germline *FANCD2* mutation, previously not reported in PanNETs. The patient underwent whole exome sequencing and single-cell RNA sequencing. Results: The patient underwent surgical treatment. We confirmed the presence of the germline mutation *FANCD2* and also detected the germline mutation *WNT10A*. The cellular composition of the PanNET was analyzed using single-cell sequencing, and the main cell clusters were identified. We analyzed the tumor genomics, and used the data to define the effect the germline *FANCD2* mutation had. Conclusions: Analysis of the mutational status of patients with PanNET may provide additional data that may influence treatment tactics, refine the plan for monitoring such patients, and provide more information about the pathogenesis of PanNET. PanNET research using scRNA-seq data may help in predicting the effect of therapy on neuroendocrine cells with *FANCD2* mutations.

## 1. Introduction

Pancreatic neuroendocrine neoplasms (PanNENs) are a heterogeneous group of rare tumors originating from neuroendocrine cells and comprising 1–2% of all pancreatic neoplasms. PanNENs are classified into functional and nonfunctional categories based on the presence or absence of hormonal hypersecretion of biologically active substances [1]. Nonfunctional PanNETs comprise about 60–90% of all PanNETs [2,3].

PanNENs are further classified into well-differentiated pancreatic neuroendocrine tumors (NETs) and poorly differentiated neuroendocrine carcinomas (NECs). NETs are divided into three grades (G1, G2, G3), depending on proliferative activity [1,4].

Over the recent decades, there has been a notable improvement in the detection of PanNETs, particularly for small nonfunctional PanNETs (sized ≤ 2 cm), due to the availability of technologically advanced high-precision imaging techniques [1,5,6,7].

Considering the malignant potential of PanNETs, surgical intervention remains the primary method of treatment. Highly differentiated nonfunctional T1 PanNETs are of particular interest, since they often follow an atypical course of progression. Some of these tumors appear clinically benign, and, therefore, in certain circumstances, they can be managed with surveillance [8]. However, some small PanNETs may exhibit a clinically aggressive course. Notably, a size of 2 cm or larger is currently considered a threshold for surgical intervention in nonfunctional PanNETs [9].

Nonetheless, any tumor would have been smaller than 2 cm at a certain point of development. Therefore, the treatment strategy for such a patient cohort remains debatable and is determined individually. According to the available data, the rate of metastatic spread to regional lymph nodes in patients with small PanNETs can reach 24–27% [10,11], with a rate of remote metastases of 7.6% [12,13].

Most PanNETs occur sporadically, and only approximately 10% of cases are associated with germline mutations as part of certain syndromes. PanNET-associated germline mutations have been reported to occur in multiple endocrine neoplasia type 1 (*MEN1*), von Hippel-Lindau syndrome (*VHL*), neurofibromatosis type 1 (*NF1*), and tuberous sclerosis complex (*TSC*) genes [14,15,16,17].

PanNETs associated with specific syndromes are typically multifocal, demonstrating diverse behavior and prognosis. In the case of germline *MEN1* mutations, nonfunctional PanNETs demonstrate malignant behavior in 13% of cases, whereas functional PanNETs are malignant in 11–50%. Metastatic progression of PanNETs does occur in a subset of patients with germline MEN1 mutations, though it is less common, as compared to sporadic PanNETs [18]. For example, liver metastases in germline *MEN1*-mutant PanNETs are identified in approximately 19% of cases, whereas in sporadic PanNETs it’s much more prevalent—in 60% of cases [19,20,21].

Germline *VHL*-mutant PanNETs are mostly isolated (67–70% of cases), demonstrating malignant behavior in 12–20% of cases [22,23,24]. PanNETs with germline *NF-1* or *TSC* mutations are extremely rare: only several such cases have been reported in the literature [18,25,26,27,28].

The most frequently described somatic mutations in sporadic PanNETs occur in the following genes: *MEN1*, *DAXX* (death domain associated protein), *ATRX* (α-thalassemia/mental retardation syndrome X-linked), and genes related to the mTOR signal pathway [14,16,17]. The available evidence suggests that *DAXX/ATRX* mutations are associated with an increased malignant potential of PanNETs. Patients with these mutations have a higher recurrence rate and lower overall survival; therefore, *DAXX/ATRX* mutations in small nonfunctional PanNETs can be considered as a potential marker of malignant progression [29,30,31,32,33,34].

Such biomarkers are particularly important for patients with small (≤2.0 cm) PanNETs when selecting between surveillance and surgical intervention. In the study conducted by Mastrosimini et al., a retrospective analysis of EUS-FNB (endoscopic-ultrasound fine-needle biopsy) data was performed to evaluate the expression of *DAXX/ATRX*, as well as alternative lengthening of telomeres (ALT) activity. The study analyzed data on 41 patients with PanNETs, among which 11 patients had small neoplasms (sized ≤ 2 cm). In 5 out of 11 patients with small PanNETs, the aggressive disease course following surgical intervention was observed, which manifested as metastatic lymph node involvement and lymphovascular invasion [33]. Another study demonstrated that recurrence-free survival of patients with small PanNETs (sized ≤ 2 cm) was lower in those with reduced *DAXX/ATRX* expression and high ALT activity. These results confirm the potential clinical significance of molecular diagnostic evaluation for pancreatic neoplasms and the importance of identifying novel predictive markers that can influence treatment strategy and patient surveillance algorithms [35].

In this article, we describe a clinical case of a small PanNET, which demonstrated tumor progression accompanied by a germline *FANCD2* mutation, previously not reported in PanNETs. The *FANCD2* gene is located on chromosome 3 and encodes a protein under the same name that is involved in DNA damage repair. Mutations in the *FANCD2* gene impair the interaction between the FANC protein complex and BRCA1 (breast cancer-associated 1). As a result, those proteins lose their capacity to prevent chromosome breakage, causing the accumulation of DNA damage.

## 2. Case Report

**Figure 1:**
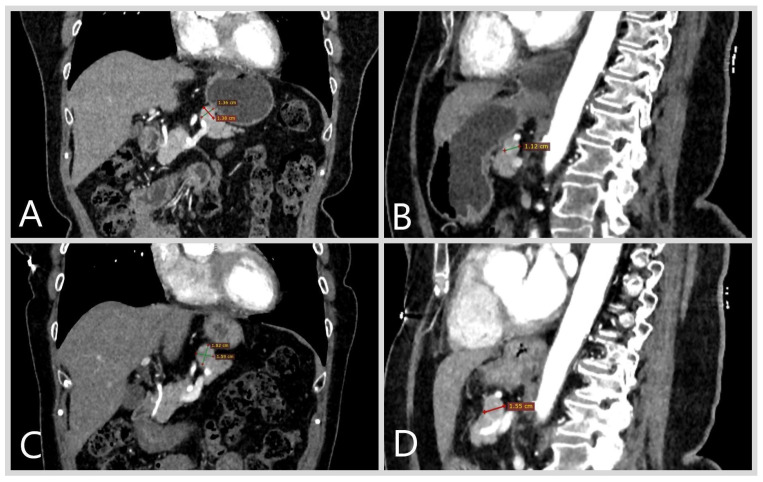
Multispiral computed tomography of the abdominal cavity, patient D. The neuroendocrine tumor of the pancreas body, with the indicated size, is highlighted with arrows. Frontal (**A**) and sagittal (**B**) views of the MSCT performed in February 2022. Frontal (**C**) and sagittal (**D**) of the MSCT performed in August 2023.

Patient D., female, aged 66 years, presented with discomfort in the epigastric area since December 2021. Upon examination at a local outpatient facility, erosive gastritis was revealed through gastroscopy. Additionally, an ultrasound of the abdominal cavity indicated the presence of a pancreatic tumor 12 × 10 mm in size. Multispiral computed tomography (MSCT), conducted in February 2022, confirmed the presence of pancreatic neoplasm up to 13 mm (Figure 1A,B). Follow-up MSCT with IV contrast enhancement in April 2022 demonstrated a pancreatic tumor of 13 × 12 mm, with a CT pattern consistent with a neuroendocrine tumor. The patient was subsequently referred to a gastroenterologist and surgeon at an expert-level institution.

The laboratory workup conducted in the autumn of 2022 did not indicate any elevation of tumor markers (Table 1). The hormonal activity of the tumor was evaluated, but no indicators of abnormal hormone production were revealed (Table 1).

The patient was diagnosed with a nonfunctional NET of the pancreatic body. The diagnosed comorbidities included grade 1, stage 1 arterial hypertension, chronic superficial gastritis, and hiatal hernia. Considering the absence of hormonal activity, small size, and lack of tumor growth over time, surveillance at a specialized expert center (every 6 months) was recommended to the patient. Follow-up MSCT in August 2023 demonstrated tumor growth, reaching a size up to 18.3 × 15 mm (Figure 1C,D).

It is known from the patient’s medical history that a germline pathogenic variant of the *FANCD2* gene was previously identified.

For our research we performed full exome NGS (next-generation sequencing) of peripheral blood lymphocytes, and identified an inactivating heterozygous *FANCD2* mutation, variant chr3:10093325T>G in the gene *FANCD2* (ENST00000675286.1, c.3888+2T>G, rs1419879344). This variant is described as pathogenic in the ClinVar and VarSome databases. To date, only one case of this specific mutation in the *FANCD2* gene has been documented in a patient with Fanconi anemia. There are currently no published data on this specific variant in patients with PanNETs [36]. Another detected germline variant was a heterozygous *WNT10A* mutation, variant chr2:218882368C>A in gene *WNT10A* (ENST00000258411.8, c.321C>A, rs121908119), described as pathogenic in ClinVar and associated with Odonto-onycho-dermal dysplasia, tooth agenesis, and other phenotypes. *WNT10A* is suggested to be a WNT signaling activator; *WNT10A* knockout results in reduced WNT signaling and β-catenin levels [37,38,39].

It should be noted that the patient has no family history of oncological disorders. Apart from PanNET, the patient had no other oncological diseases during her life.

Taking into account the tumor growth by more than 5 mm over 1 year of follow-up, along with the presence of an inactivating mutation with malignant potential, the decision was made to proceed with surgical intervention. Laparoscopic tumor enucleation was performed in December 2023. The post-surgical period was unremarkable.

Further follow-up for this patient was recommended: tests for chromogranin A, serotonin, gastrin serum level-3, 6 and 12 months after surgery; CT scan of chest, abdomen, and pelvis 6 and 12 months after surgery; PET/CT-Ga-68-DOTA-TATE in case of clinical, laboratory, or radiological signs of progression.

**Figure 2:**
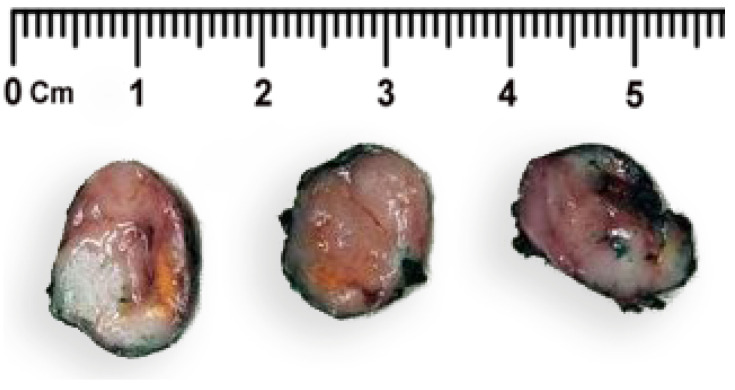
Gross presentation of the removed neoplasm section, the surgical resection margin is marked in green ink.

The pathomorphological examination of the resected fragment of the pancreatic tissue (Figure 2) demonstrated a round s-sped tumor in a thin fibrous capsule.

**Figure 3:**
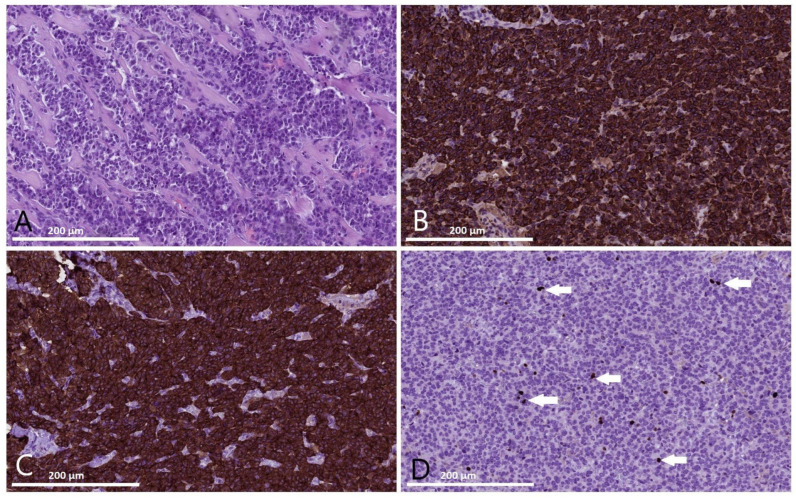
Histological and immunohistochemical examinations of the pancreatic surgical specimen from patient D. (**A**) The pancreatic neuroendocrine tumor demonstrates trabecular and organoid-like structure, consisting of mid-sized epithelioid cells with monomorphic round-shaped nuclei with typical microvesicular chromatin (hematoxylin-eosin staining, magnitude ×400). (**B**) Strong diffuse cytoplasmatic staining of tumor cells with anti-chromogranin A antibody. (**C**) Strong diffuse cytoplasmatic staining of tumor cells with anti-synaptophysin antibody. (**D**) Ki67, the marker of proliferative activity, is positive in 2% of tumor cell nuclei (arrows).

The tumor was histologically organized as trabecular and organoid-like (pseudoglandular) structures (Figure 3A). These structures were composed of middle-sized epithelioid cells with eosinophilic cytoplasm and monomorphic round-shaped nuclei containing typical microvesicular chromatin, without evidence of mitotic activity (no mitosis was found over the area of 10 mm^2^). The tumor focally grew through its fibrous capsule, with initial invasion into the adjacent pancreatic tissue. There was no evidence of invasion into lymph vessels or perineural growth. An area of sclerosis was found in the center of the neoplasm. No necrosis was observed. Tumor cells were located less than 0.1 mm from the marked resection margin; the adjacent pancreatic tissue demonstrated findings consistent with significant coagulation.

**Figure 4:**
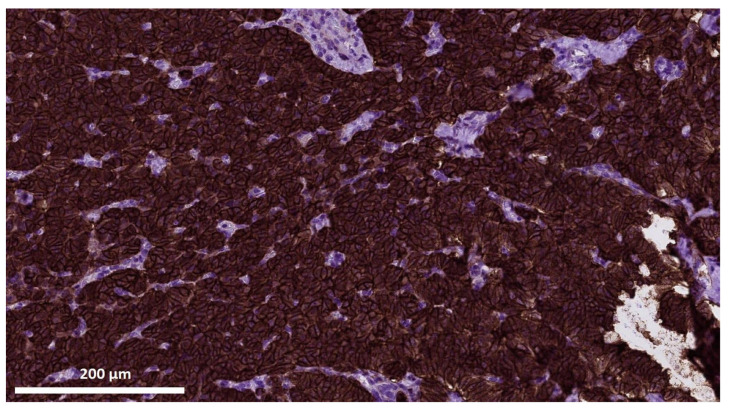
Significant membrane staining of 100% of tumor cells for SSTR2 as part of the immunohistochemical examination of the pancreatic surgical specimen from patient D. (magnification ×400).

An immunohistochemical (IHC) examination was performed to confirm tumor histological type and determine its proliferative activity. The IHC analysis demonstrated a positive cytoplasmatic reaction of the tumor cells to staining with neuroendocrine markers chromogranin A (Figure 3B) and synaptophysin (Figure 3C). Ki67, the marker of proliferative activity (Figure 3D) was positive in 2% of nuclei of the tumor cells. In addition, 100% of the membranes of the tumor cells stained positive for SSTR2 (Figure 4), which, according to the criteria proposed by Volante M. et al. [40], is typical for level 3+ expression of type 2 somatostatin receptors. Therefore, a well-differentiated pancreatic neuroendocrine tumor (NET, G1) was pathologically verified, with a size of 1.9 cm, pT1 pNx L0V0 Pn0.

**Figure 5:**
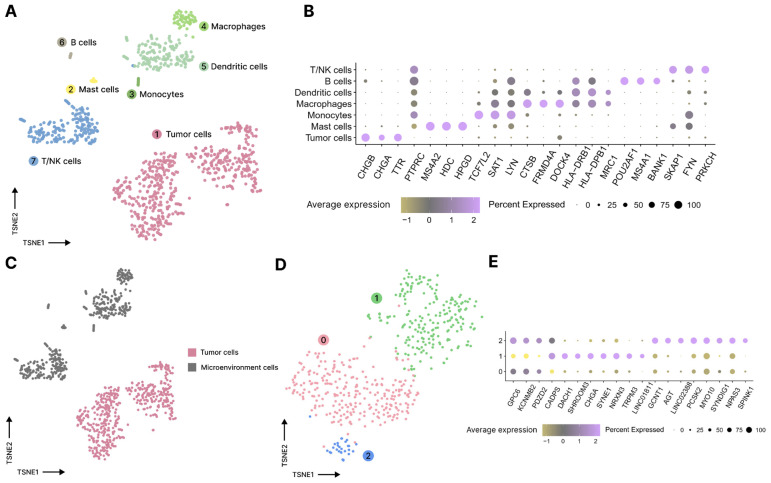
ScRNA-seq analysis of the tumor sample. (**A**). Annotated cells on t-SNE coordinates. (**B**). A dot plot with cell type markers used for annotation. (**C**). Cells on t-SNE coordinates from A, annotated based on the result of CNV analysis of tumor clonality. (**D**). Clustered neuroendocrine tumor cells on t-SNE coordinates. (**E**). Main markers of tumor cell clusters.

A piece of the collected surgical specimen of the tumor tissue was used to prepare a single-cell suspension for subsequent single-cell RNA-sequencing (scRNA-seq) library preparation according to the 5′ 10× Genomics protocol. After processing and quality control, the resulting Seurat object contained 954 high-quality cells. After dimensional reduction and clustering, cells were manually annotated into tumor cells and immune cells, namely, mast cells, monocytes, macrophages, dendritic cells, B cells, and T/NK cells, based on the results of differential expression analysis and the known cell type markers (Figure 5A,B). The copy number variation (CNV) analysis revealed that all neuroendocrine cells were annotated as tumor cells and belonged to one CNV clone despite the observed intratumoral transcriptomic heterogeneity (Figure 5C–E). This might be due to distinct spatial localization of cells in tumor tissue or differences in point mutations in neuroendocrine cells. Differential expression analysis between tumor clusters revealed distinct expression profiles in clusters 1 and 2, while cluster 0 had only a few specific marker genes. KEGG pathway analysis using gene set enrichment analysis (GSEA) revealed no relevant pathways enriched in any of the tumor cell populations.

**Figure 6:**
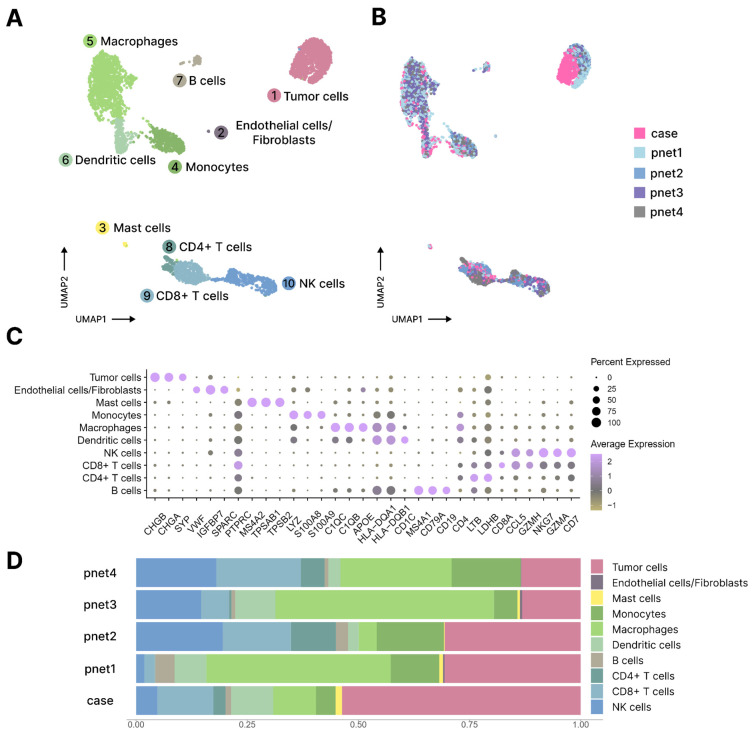
Integration and comparison of the obtained scRNA-seq data on PanNET with published scRNA-seq data. (**A**). An integrated and annotated cells’ representation on UMAP coordinates. (**B**). Distribution of cell sources on UMAP coordinates (pnet1–4 are samples from the published dataset). (**C**). A dot plot with cell type markers used for annotation of integrated data. (**D**). Distribution of cell types in samples (pnet1–4 are samples from the published dataset).

In order to define the components of the tumor microenvironment more precisely and characterize its compositional patterns, we integrated our dataset with published scRNA-seq data from four samples of PanNETs obtained using the 10× Genomics 5′ library preparation protocol (Figure 6A) [41]. After batch correction, the final cell populations comprised cells from different samples (Figure 6B). The cell type labels were manually assigned based on known marker genes (Figure 6C). Integration with published data enabled the division of T/NK cells into CD4+ T cells, CD8+ T cells, and NK cells, and the identification of the population of endothelial cells/fibroblasts although it was not present in the piece of sample used for scRNA-seq analysis (Figure 6D).

Overall, we observed similar proportions of different cell types in all samples with a higher share of stromal cells in tumors from published scRNA-seq data due to the presence of a CD45-positive cell enrichment step in the experimental design.

However, to determine associations between *FANCD2* and *WNT10A* germline mutations and clinical manifestation, e.g., response to therapy and prognosis or tumor molecular-level alterations, there is a need for a group of patients harboring variants in the gene. The small amount of published data on PanNETs prevents a more complex analysis; however, the scRNA-seq data and results of basic characterization of the tumor sample presented in this work can enhance future studies.

**Figure 7:**
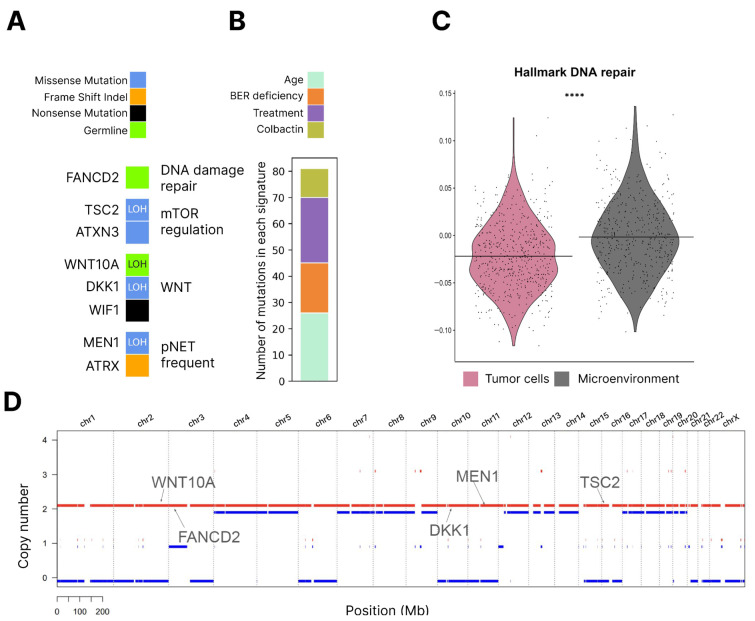
(**A**). Somatic and germline mutations in the tumor sample. (**B**). COSMIC SBS mutational signatures. (**C**). Hallmark DNA repair signature score in the patient’s tumor cells (pink) vs. microenvironment cells (grey), **** *p*-value < 0.0001, Wilcoxon rank sum test. (**D**). Somatic CNA profile. Lines represent mean values of the score.

To further assess the impact of germinal *FANCD2* and *WNT10A* mutations on the tumor, we conducted WES (whole exome sequencing) for the patient’s tumor sample. We discovered that the tumor gained a 2.05 mutation/Mb TMB (tumor mutational burden). While this rate would be generally considered a low TMB, for PanNET, this rate is notably high as the mean TMB for PanNET is demonstrated to be 0.82 (range 0.04–4.56) or lower [42,43]. This, indeed, might be a consequence of impaired DNA repair with *FANCD2* mutation. Additionally, we decomposed the sample mutational profile to COSMIC mutational signatures and discovered the presence of SBS30, which is associated with base excision repair deficiency (Figure 7B) [44,45]. As of now, *FANCD2* has been connected to more than DNA interstrand crosslink (ICL) repair: it is involved in the binding of various DNA damage repair factors to DNA in a number of mechanisms, resolves conflicts between replication and transcription events, and participates in mRNA nuclear export to prevent R-loops [46,47,48]. It is possible that in the PanNET setting, *FANCD2* damage has a more pronounced effect on base excision repair (BER) mechanisms than on others. However, it should be noted that the tumor also has a deletion of *MUTYH* (2 copies out of 4 deleted, LOH). *MUTYH* damage has been previously connected with BER deficiency [49], which might be an alternative explanation for the presence of a BER deficiency signature.

To investigate the further effect of *FANCD2* variant on genome stability, we estimated microsatellite instability score (MSI-score) and homologous recombination deficiency (HRD-score) for the tumor, and the latter was suggested to be especially indicative due to *FANCD2* participation in homologous recombination. MSI-score was equal to 1.9% of the inspected sites and HRD-sum reached 27. While we do not have an MSI or HRD PanNET cohort to draw comparisons, it can be reasoned that the sample is MSI-stable due to the very low percentage of altered sites. However, the HRD score, when compared with HRD values in breast cancer, while it does not reach levels of BRCA-deficient samples, is still higher than 40–50% of samples without BRCA deficiency. It can be carefully suggested that the obtained value can be considered high for less aggressive tumor types, such as PanNETs. We also called fusions from the WES data and discovered no targetable fusions in this sample.

Nevertheless, the high mutation rate alone can still be connected with impaired DNA damage repair due to *FANCD2* damage. Additionally, it has been proposed that *FANCD2* may protect the genome from unrestricted resection by DNA2, which results in chromosomal rearrangements and tumor aneuploidy [50]. It is worth noting that our case has a decent number of these CNA events (Figure 7D). On the other hand, the variant allele frequency (VAF) of this variant in the tumor was 0.26, indicating that one of the copies with the *FANCD2* pathogenic variant was lost. Additional evidence of DNA damage repair impairment was a comparison of signature activity between the microenvironment and tumor cells of the patient (Figure 7C). DNA damage repair activity was decreased in tumor cells (*p*-value < 0.0001). Unfortunately, direct comparison with signature activity in tumors without *FANCD2* mutation was impossible due to the prevalence of the batch effect. Still, the presented evidence speaks of a possible influence of *FANCD2* mutation on tumor processes.

*WNT10A* heterozygous germline variant was present in the tumor sample with VAF 0.15, which corresponded to the loss of the *WNT10A* pathogenic variant in the tumor sample (as predicted purity is 0.68 and there is LOH in the *WNT10A* region (Figure 7D)). Interestingly, the tumor instead possessed two somatic variants in negative WNT regulators, genes *DKK1* and *WIF1* (Figure 7A) [51,52], while WNT10A damage or knockout was connected with WNT deactivation [37,38,39]. We suggest that an increase in WNT signaling is beneficial for this PanNET tumor, hence the loss of the WNT-inhibiting variant and mutations in WNT-negative regulators. However, it is impossible to make signature activity comparisons with microenvironment cells due to their own active WNT signaling and proliferation.

In addition, the tumor harbored a number of PanNET common somatic mutations, like mutations in mTOR signaling, including *TSC2*, *MEN1*, and *ATRX* mutations (Figure 7A), perturbing chromatin modeling pathways. These somatic mutations are commonly associated with a more aggressive tumor profile and worse prognosis [30,31,53]. The CNA profile also possesses traits of an aggressive tumor subtype (termed Group 1 in Lawrence et al.) [43], namely, aneuploidy of chromosome 11 and aneuploidy of other chromosomes, which results in loss of heterozygosity. While, with a single case report, it is impossible to directly connect *FANCD2* deficiency with these features, we can carefully suggest that deficient DNA damage repair allows for more frequent and variable mutation occurrence and, thus, a capability to evolve and gain variants for further propagation more quickly.

## 3. Discussion

Currently, the role of *FANCD2* in tumors seems dual. Germline *FANCD2* mutations were detected in patients with various tumors and were suggested to be procarcinogenic [54,55]. *FANCD2*-low cell lines established from ovarian surface epithelial cells demonstrated chromosomal breakage response to mitomycin C, which demonstrates that *FANCD2*-low cells might be more susceptible to tumorigenic processes [56]. Additionally, recently, somatic *FANCD2* mutation (c.2022-5C>T) emerged as a biomarker of early progression in chronic myeloid leukemia. This mutation was suggested to be damaging and inactivating [57]. Among neuroendocrine neoplasms of various locations, *FANCD2* mutation was detected in rectal neuroendocrine neoplasms and intestinal NETs [58,59]. However, the occurrence was not high (5/38 and 1/48) and those mutations were not investigated in detail. In one out of thirteen patients with pancreatic neuroendocrine neoplasms, Shunrong et al. detected a mutation at the *FANCD2* splicing site. Clinically, that patient was found to have a PanNET G2 T3N1M1a with liver metastasis and metastasis in a regional lymph node [60].

However, *FANCD2* expression was repeatedly reported to be associated with poor prognosis in various cancers and to be higher in tumors than in normal tissues. Zhao et al. [61] demonstrated this on TCGA data; the same results were achieved with testing *FANCD2* expressions or staining in breast cancer [62,63], endometrial carcinoma [64], colorectal cancer [65], esophageal squamous cell cancer [66], hepatocellular cancer [67], lung adenocarcinoma [68], and in pan-cancer settings [69]. The only outlier is in breast cancer research by Rudland et al., where the absence of FANCD2 staining was associated with poor survival [70]. In most of these research studies (notably, except for Rudland et al. [70]), elevated levels of FANCD2 were strongly associated with increased proliferation (G2M signatures, Ki67). This might explain some of the risks connected with FANCD2 as an increase in Ki67 levels is universally connected with poor survival of tumor patients [71]. For example, Zhao et al. [61] did not include proliferation in their survival analysis. However, in breast cancer, FANCD2 staining was demonstrated to be predictive independent of Ki67 [62,63]. This effect on patient survival might be due to FANCD2 resolving replication fork stalling (thus correlating with the intensity of proliferation) and conferring resistance to chemotherapy and chromosomal breaks [56,57,61]. Without FANCD2, tumor proliferation stalls; however, early on in tumor development, its damage might provide genomic instability required for tumor development and evolution.

The presence of a *FANCD2* mutation probably contributes to the potential initiation of the tumorigenesis process, also contributing to the progression of a pre-existing malignancy, thereby increasing the metastatic potential of a tumor. In this clinical case, the tumor growth of more than 5 mm within a year is noteworthy and may be due to the presence of a germline *FANCD2* mutation, which, along with other considerations, eventually determined subsequent treatment strategy for this patient, resulting in surgical intervention despite the small size of the tumor.

Pre-surgical identification of germline mutations in patients with PanNETs may become a decisive consideration when selecting individual treatment strategies in a patient with stage T1 PanNET.

The significance of pathogenic variants in *FANCD2* in tumors for therapy choice is also worth consideration. It stands to reason that, since *FANCD2* is required for tumor survival and DNA ISL repair, patients with mutated *FANCD2* might benefit from PARPi (PARP inhibitors) [72,73]. While this patient is unlikely to require PARPi due to the tumor type and current absence of metastatic process, it is of interest for other patients and tumor types whether *FANCD2* mutations should be routinely tested as a PARPi biomarker. Previously, it has been demonstrated by Fallah et al. [74] that, while patients with variants in *BRCA1, BRCA2, CDK12*, and *PALB2* benefitted from PARPi, there was no significant benefit for patients with variants in *CHK2* and *AT*, although both of these genes are participants of HR repair. While some clinical trials have tried testing for larger lists of Fanconi anemia group genes, they have not detected any *FANCD2* variants in their patients [75], so their value is currently unresolved. In this study, the *FANCD2* variant was demonstrated to have an effect on tumor landscape compared to PanNETs without this mutation. However, more cases, especially of tumor types that commonly benefit from PARPi, should be analyzed before judging *FANCD2* pathogenic variants to be valuable biomarkers for this treatment.

An examination of the tumor specimen using scRNA-seq data may aid in predicting the effect of the therapy on *FANCD2*-mutant neuroendocrine cells as well. In the present study, scRNA-seq analysis helped determine various cellular populations of the microenvironment and describe heterogeneity inside the tumor. Three distinct clusters of tumor cells were characterized by different expression profiles, despite all belonging to the same CNV clone. Expression of genes involved in DNA damage repair was lower in the tumor component in comparison to the microenvironment cells, demonstrating the impairment of that process in neuroendocrine cells caused by the *FANCD2* mutation and, presumably, resulting in a relatively high TMB in this specimen and dynamic growth over the 1 year period of follow-up. Tumor scRNA-seq data are publicly available through Gene Expression Omnibus and can be used for a more detailed analysis of PanNETs in larger cohorts.

### Limitations

A limitation of our study is that it is a report on only one patient with PanNET. Naturally, a larger sample of patients with this pathology is required to confirm the role of *FANCD2* in the genesis of PanNET. Another limitation is the small selection of scRNA-seq PanNET samples in the literature, which complicates comparative analysis. The batch effect also complicates comparisons with samples without *FANCD2* mutation.

## 4. Materials and Methods

### 4.1. Sources of the Human Tissue and Blood Samples

The human tissue sample of the pancreatic neuroendocrine tumor and blood samples involved in this study were obtained from the Moscow Clinical Scientific Center named after Loginov, Moscow 111123, Russian Federation. This study was approved by the Local Ethics Committee of the Moscow Clinical Scientific Center named after Loginov of the Moscow Department of Healthcare.

### 4.2. Histopathology and Immunohistochemistry

Tissue samples were collected, fixed, and then paraffin-embedded. A total of 5 µm sections were obtained and subjected to histopathology and immunohistochemistry (IHC) analyses. We used hematoxylin and eosin (HE) staining for histopathological examination and immunohistochemistry for expression analysis, respectively. IHC was performed on a fully automated VENTANA Benchmark XT stainer (VENTANA Medical Systems; Roche Group, Tucson, AZ, USA) using the following antibodies: anti-synaptophysin (Kit-0022; MXB Biotechnologies, Fuzhou, China), anti-chromogranin A (MAB-0707; MXB Biotechnologies, Fuzhou, China), anti-Ki67 (IR626; Dako Products, Santa Clara, CA, USA), and anti-somatostatin R2 (EP149, Abcam, Massachusetts, MA, USA), together with the Optiview DAB IHC detection and Optiview amplification kits (VENTANA Medical Systems; Roche Group, Tucson, AZ, USA). Images were taken using digital pathology scanners: Aperio AT2 (Leica Biosystems, Wetzlar, Germany) and NanoZoomer S360 (Hamamatsu Photonics, Shizuoka, Japan). Ki67 automated counts were measured in tumor “hotspots” using QuPath software, version 0.4.0.

### 4.3. Preparation of a Single-Cell Suspension, 10× Library Preparation and Sequencing

Fresh tumor tissues were mechanically chopped with scalpels on a plate and enzymatically digested with collagenase type IV (Servicebio, Wuhan, Hubei, China) and DNAse I (Magen, Guangzhou, Guangdong, China). Following digestion, the cell suspension was subjected to erythrocyte removal using the red blood cell lysis buffer (QuantoBio, Beijing, China). The cell suspension was filtered through a 70 μm strainer (MACS, Bergisch Gladbach, Germany), and dissociated cells were pelleted and re-suspended in phosphate buffer saline (PBS) with 0.04% BSA (bovine serum albumin). The Chromium Next GEM Single Cell 5′ v2 Kit (10× Genomics, Pleasanton, CA, USA) and the Chromium Single Cell Controller Instrument were used to generate a 10× single-cell library according to the manufacturer’s instructions. After the library construction and quality control, sequence data were generated using SurfSeq 5000, and 377 million reads were obtained.

### 4.4. Single-Cell Transcriptome Data Preprocessing and Analysis

The raw reads in the fastq format were processed using the Cell Ranger pipeline (version 7.1.0) and the retrieved count matrix contained 1376 cells with a median of 1046 genes and 1716 UMIs detected per cell. Preprocessing and downstream analysis of scRNA-seq data were performed in RStudio (R version 4.3.3). Quality control included the removal of cells with inappropriate gene–umi relationships and a low or high number of unique RNA molecules by gene.vs.molecule.cell.filter, the pagoda2 package [76], high mitochondrial (>20%) or ribosomal (>20%) RNA content, and droplets, containing multiplets (scrublet package, version 0.2.3 [77]). The expression profile was corrected for ambient RNA using DecontX [78] (celda package, version 1.16.1), and cells containing >500 UMIs and >250 unique genes were retained. Basic steps of downstream data processing including normalization (LogNormalize), scaling (ScaleData), search for variable features (FindVariableFeatures, nfeatures = 2000), dimensional reduction (RunPCA, RunUMAP, RunTSNE, dims = 20), clustering (FindClusters, FindNeighbors), and visualization were performed using standard Seurat package functions. The remaining 954 high-quality cells with a median of 2081 unique RNA molecules and 1281 unique genes per cell in the final Seurat object were manually annotated by performing differential expression analysis with FindAllMarkers function (test.use = “roc”) and searching for known cell types’ markers in PanglaoDB [79] and The Human Protein Atlas proteinatlas.org. Published scRNA-seq data of four PanNETs [41] was obtained from the Broad Institute Single Cell Portal (https://singlecell.broadinstitute.org/single_cell (accessed on 20 October 2024)). Normalized and filtered count matrices were processed using basic steps, including the removal of cells with a low number of unique genes (nFeature_RNA < 500), scaling, search for variable features (nfeatures = 3000), dimensional reduction (dims = 20), and clustering. Integration with published data was performed using canonical correlation analysis (CCA) implemented in Seurat (nfeatures = 3000, dims = 20). Annotation was performed as previously described using differential expression analysis (FindAllMarkers, test.use = “roc”) and search for known marker genes.

### 4.5. Hallmark Signature Scoring

Hallmark gene signatures were obtained from the Molecular Signature Database implemented in the msigdbr R package (version 7.5.1) [80]. Each cell was assigned a score using the AddModuleScore function from the Seurat package. The significance of differences in scores in tumor versus microenvironment cells was evaluated using the Wilcoxon rank sum test.

### 4.6. Whole Exome Sequencing and Analysis

WES of tumor and blood samples was prepared with VAHTS Universal Plus DNA Library Prep Kit for Illumina V2, VAHTS Target Capture Core Exome Panel with VAHTS Target Capture Hybridization, and Wash Kit were used in hybridization. Prepared libraries were sequenced with SurfSeq5000.

Raw fastq reads were trimmed with fastp (version 0.23.2) and aligned with BWA (version 2.2.1) to the GRCh38 genome. The mean coverage of blood and tumor samples (mosdepth, version 0.3.3) was 253× and 513×, respectively. DeepVariant (version 1.5.0) was used for germinal mutation calling from blood samples and EnsembleVEP (version 111.0) was used for variant annotation. For paired somatic calling, Manta and Strelka (versions 1.6.0 and 2.9.10) were used, and then, only variants with FILTER = PASS were annotated with EnsembleVEP (version 109.3) and processed with the vcf2maf package (version 1.6.22). All variants were filtered with the following thresholds: TumorVAF > 0.05, NormalVAF < 0.05, tumor depth > 20, tumor alt count > 10, and normal depth > 10. Only variants passing these filters were used for further TMB and mutational signature calculation.

Mutational signatures were calculated with SignatureProfilerAssignment (version 0.1.8) [81] with default parameters. Cosmic signatures v. 3.4. were used. Fusions were called with FuSeqWES (version 1.0.0) [82]. CNA was called with sequenza-utils (version 3.0.0) and R package sequenza (version 3.0.0) [83]. Pileups for sequenza were generated with samtools (version 1.17) in WES-covered regions in positions of variants that have population AF > 0.05 from dbSNP. GRCh38 genomic annotation was intersected with a segment file to get gene-level copy numbers.

MSI-scores were calculated with msisensor-pro (version 1.3.0) [84]. HRD-scores were calculated with the scarHRD package [85].

### 4.7. scRNA-Seq CNV Analysis

The search for somatic copy number variations and separation between normal and malignant cells in scRNA-seq data was conducted using Numbat in accordance with the vignette [86]. The analysis was enhanced by the use of WES bam files on the preprocessing step (population SNP pileup and phasing).

## Figures and Tables

**Table 1:** Case laboratory values.

Test	Result	Normal Range
CA 19-9 (cancer antigen 19-9)	9.89 U/mL	<37 U/mL
CEA (carcinoembryonic antigen)	3.4 ng/mL	<5.0 ng/mL
AFP (alpha-fetoprotein)	2.26 ng/mL	0–7 ng/mL
gastrin	59.0 ng/L	13–115 ng/L
chromogranin A	41.0 ng/mL	0–100 ng/mL
calcitonin	0.71 ng/L	0.0–6.40 ng/L
serotonin	7.93 µmol/L	1.85–8.16 µmol/L
ACTH (adrenocorticotropic hormone)	5 pg/mL	0–46 pg/mL

## Data Availability

The original scRNA-seq data presented in the study are openly available on Gene Expression Omnibus under accession number GSE279805.

## Abbreviations

ACTH	adrenocorticotropic hormone
AFP	alpha-fetoprotein
ALT	alternative lengthening of telomeres
ATRX	α-thalassemia/mental retardation syndrome X-linked protein
BER	base excision repair
BRCA1	breast cancer-associated 1 protein
CA 19-9	cancer antigen 19-9
CCA	canonical correlation analysis
CEA	carcinoembryonic antigen
CNV	copy number variation
COSMIC	catalogue of somatic mutations in cancer
DAXX	death domain-associated protein
EUS-FNB	endoscopic-ultrasound fine-needle biopsy
GSEA	gene set enrichment analysis
HR	homologous recombination
HRD-score	homologous recombination deficiency score
ICL	interstrand crosslink
IHC	immunohistochemical
KEGG	Kyoto encyclopedia of genes and genomes
LOH	loss of heterozygosity
*MEN1*	multiple endocrine neoplasia type 1 gene
MSI-score	microsatellite instability score
MSCT	multispiral computed tomography
NGS	next-generation sequencing
NECs	neuroendocrine carcinomas
*NF1*	neurofibromatosis type 1 gene
PanNENs	pancreatic neuroendocrine neoplasms
PanNETs	pancreatic neuroendocrine tumors
SSTR2	somatostatin receptor type 2
scRNA-seq	single-cell RNA-sequencing
TCGA	The Cancer Genome Atlas
TMB	tumor mutational burden
*TSC*	tuberous sclerosis complex gene
UMI	unique molecular identifier
VAF	variant allele frequency
*VHL*	von Hippel-Lindau syndrome gene
WES	whole exome sequencing
